# Does phenotyping of *Hypericum* secondary metabolism reveal a tolerance to biotic/abiotic stressors?

**DOI:** 10.3389/fpls.2022.1042375

**Published:** 2022-11-30

**Authors:** Katarína Bruňáková, Miroslava Bálintová, Linda Petijová, Eva Čellárová

**Affiliations:** Department of Genetics, Institute of Biology and Ecology, Faculty of Science, Pavol Jozef Šafárik University in Košice, Košice, Slovakia

**Keywords:** Phenolic compounds, proline, carotenoids, CAT, SOD, APX, cold stress

## Abstract

In this review we summarize the current knowledge about the changes in *Hypericum* secondary metabolism induced by biotic/abiotic stressors. It is known that the extreme environmental conditions activate signaling pathways leading to triggering of enzymatic and non-enzymatic defense systems, which stimulate production of secondary metabolites with antioxidant and protective effects. Due to several groups of bioactive compounds including naphthodianthrones, acylphloroglucinols, flavonoids, and phenylpropanes, the world-wide *Hypericum perforatum* represents a high-value medicinal crop of *Hypericum* genus, which belongs to the most diverse genera within flowering plants. The summary of the up-to-date knowledge reveals a relationship between the level of defense-related phenolic compounds and interspecific differences in the stress tolerance. The chlorogenic acid, and flavonoids, namely the amentoflavone, quercetin or kaempferol glycosides have been reported as the most defense-related metabolites associated with plant tolerance against stressful environment including temperature, light, and drought, in association with the biotic stimuli resulting from plant-microbe interactions. As an example, the species-specific cold-induced phenolics profiles of 10 *Hypericum* representatives of different provenances cultured *in vitro* are illustrated in the case-study. Principal component analysis revealed a relationship between the level of defense-related phenolic compounds and interspecific differences in the stress tolerance indicating a link between the provenance of *Hypericum* species and inherent mechanisms of cold tolerance. The underlying metabolome alterations along with the changes in the activities of ROS-scavenging enzymes, and non-enzymatic physiological markers are discussed. Given these data it can be anticipated that some *Hypericum* species native to divergent habitats, with interesting high-value secondary metabolite composition and predicted high tolerance to biotic/abiotic stresses would attract the attention as valuable sources of bioactive compounds for many medicinal purposes.

## 1 Introduction

To resist the impact of a stressful environment, plants evolved adaptation strategies based either on avoidance or tolerance response (Koch et al., 2016). While the avoidance strategy enables the plant to minimize negative effects of a certain stress, the tolerance mechanism relies on the ability to withstand the unfavorable conditions (Puijalon et al., 2011). In response to bacterial, fungal, and viral diseases, or insect and herbivores attacks plants restrict the pathogen multiplication or minimize the effect of infection, or tissue damage (Pagán and García-Arenal, 2018). Similarly, plants adapt to adverse abiotic conditions like freezing (Körner, 2016), drought (Yang et al., 2020), high salinity (Soliman et al., 2018), or mechanical stress (Puijalon et al., 2011) through the tolerance or avoidance mechanisms.

To reveal the underlying relations between different adaptation strategies, the combined genotypic and phenotypic data are usually applied to characterize the patterns of plant adaptation to local environments (Gao et al., 2021). The biochemical markers including secondary metabolites (SMs), phytohormones, or proteins, along with morphological and anatomical traits like plant shape, leaf structure or area, and physiological parameters, such as chlorophyll content, electrolyte leakage, or water potential are usually used as indicators of the biotic/abiotic stresses (Kosakivska, 2008; Fernandez et al., 2016; Soltabayeva et al., 2021).

Even though the ability of plants to adapt to a certain stress requires a unique response leading to appropriate physiological and metabolic alterations, the overall plant defense is associated with an intensive oxygen metabolism generating reactive oxygen species (ROS) including radicals, especially superoxide anion (O_2_^•-^), hydroxyl radical (OH^•^), and non-radical molecules, such as singlet oxygen (^1^O_2_), or hydrogen peroxide (H_2_O_2_), as well as reactive nitrogen species (RNS), particularly nitric oxide (NO), and NO-derived molecules (You and Chan, 2015; Sewelam et al., 2016). The reactive molecules are produced in plant tissues as by-products of normal cell metabolism and have a dual role in many biological processes; although the ROS and RNS are associated with oxidative damage of tissues, they act as important signals to activate the stress response (Farnese et al., 2016; Kerchev and Van Breusegem, 2022). Spreading a stress signal from the local tissues to entire plant, the ROS waves were recently referred to be required for plant adaptation to abiotic stresses including light or temperature (Zandalinas et al., 2020).

In relation to stress signalization, the ROS and RNS are involved in promoting the acquired systemic plant response comprising: i) the systemic acquired resistance (SAR) initiated by interactions of cells with pathogens (e.g., viruses, bacteria, or fungi), ii) the systemic acquired acclimation (SAA) to abiotic stimuli, such as extreme temperature, irradiation, osmotic stress, or salinity, and iii) the systemic wound response to mechanical stress induced by various biotic or abiotic stimuli (Mittler and Blumwald, 2015). However, in the complex stress signaling network, many other components including calcium (Ca^2+^), calmodulin (CaM), G-proteins, the plant hormones, and stress-signal molecules like abscisic acid (ABA), salicylic acid (SA), jasmonic acid (JA), and ethylene, the mitogen-activated protein kinases (MAPKs), and transcription factors (TFs) are involved (Sewelam et al., 2016).

When the overproduction of ROS under stress conditions exceeds the cellular scavenging potential, the oxidative destruction of macromolecules, such as lipids and proteins, negatively impacts cellular metabolism leading to changes of membrane fluidity and ion transfer, loss of enzyme activities, inhibition of protein synthesis, damage of cellular components, and activation of a programmed cell death (Kumari et al., 2021). To minimize the oxidative injury, plants possess enzymatic and non-enzymatic antioxidants (AOXs) (Sharma et al., 2012). The ROS detoxifying proteins, such as superoxide dismutase (SOD), catalase (CAT), and enzymes of ascorbate-glutathione (AsA-GSH) cycle, ascorbate peroxidase (APX) or glutathione reductase (GR), represent the main components of the enzymatic AOXs. The ROS-scavenging enzymes were shown to be involved in maintaining plant homeostasis (Caverzan et al., 2016), as well as in ROS signaling (Miller et al., 2010). For example, the deficiency in enzymatic AOXs leads to overproduction of radical molecules, which in turn act as the signal enhancing the plant tolerance to a certain stress (Miller et al., 2007; Vanderauwera et al., 2011; Sachdev et al., 2021). Non-enzymatic antioxidants include the products of plant secondary metabolism, like phenolic compounds, alkaloids, carotenoids, tocopherols, along with small-molecules, such as ascorbate (AsA), glutathione (γ-glutamyl-cysteinyl-glycine, GSH), proline and other amino acids (Latowski et al., 2014; Ashraf et al., 2019; Jan et al., 2021a). The physiological function of SMs in defense responses against abiotic stresses and pathogens, as well as mediation of the interactions between a plant and other organisms, is well documented (Fang et al., 2011; Li et al., 2020; Pang et al., 2021). The accumulation of SMs with antioxidant activities enhance the overall stress tolerance; it has been shown that plants, which are tolerant to oxidative stress exhibit also tolerance to various environmental conditions, such as extreme temperature, drought, salinity, pathogens, and combinations of them (Sewelam et al., 2014; Nguyen et al., 2018; Nadarajah, 2020).

Within the extremely diverse genus *Hypericum* comprising nearly 500 species, the St. John's wort – *Hypericum perforatum* – represents the most renowned and investigated species (Coste et al., 2021). *H. perforatum* is a medicinal plant traditionally used for treatments of depression and other neurological disorders. The *Hyperici herba* contains a lot of biologically active constituents; phenolic compounds including naphthodianthrones (hypericin and pseudohypericin), phloroglucinol derivatives (hyperforin, adhyperforin), and flavonoids, representing well-known and frequently analyzed SMs. Showing a range of bioactivities, the phenolic compounds were distinguished as valuable phytochemicals for a broad spectrum of pharmaceutical applications (Agapouda et al., 2019). In the modern medicine, hypericins are promising photosensitizers applied in photodynamic diagnosis and therapy of cancer (Kleemann et al., 2014; Theodossiou et al., 2017). Due to inhibitory effect on the growth of microorganisms, the naphthodianthrone compounds are also effective in plant protection against a number of pathogenes (Zambounis et al., 2020; Sytar et al., 2021). The polycyclic polyprenylated acylphloroglucinols (PPAPs) are responsible for most of *H. perforatum* biological activities, including antidepressant, anti-inflammatory, and antimicrobial effects (Bridi et al., 2018). Flavonoids, especially the flavonols quercetin, kaempferol and their glycosides, contribute to antioxidant properties of *H. perforatum* alcoholic extract (Makarova et al., 2021). Melatonin (N-acetyl-5-methoxytryptamine) is other important bioactive compound of *H. perforatum* (Zhou et al., 2021), which has many physiological functions in humans. This molecule is involved in the sleep-wake cycle or the circadian rhythm regulation (Zisapel, 2018), interacts with the immune system (Ma et al., 2020), protects an organism against oxidative stress (Manchester et al., 2015; Galano and Reiter, 2018), exhibits the antidepressant effects (Wang et al., 2022), and is involved in the nerve regeneration processes (Stazi et al., 2021).

Based on the state of the art we discuss the alterations of *Hypericum* spp. phenolics content in relation to various abiotic and biotic stresses (section 2) with an emphasis on determination of the predictive value of phenolic compounds and other AOXs in revealing the freezing resistance strategy of *Hypericum* plants which is illustrated in the case study (section 3). We show that phenotyping of phenolics composition, especially flavonoids might contribute to revealing the freezing tolerance/avoidance strategy in the genus *Hypericum*. As summarized in section 4, several conclusions were made regarding the changes in *Hypericum* secondary metabolism in relation to overall stress tolerance.

## 2 Phenotyping of secondary metabolism in response to biotic/abiotic stresses in *Hypericum* spp.

The genetically predetermined plant metabolite profile is modulated by various external and internal stimuli. Usually, the accumulation of SMs is related to the plant developmental stage and often requires the presence of specialized cells or cell structures (Isah, 2019). The phytochemical composition also depends on the crosstalk between plant and its microbiome (Pang et al., 2021), and is modified by environmental conditions like the intensity of light, extreme temperatures, drought, or salinity (Yang et al., 2018; Wasternack and Strnad, 2019; Li et al., 2020). The stress-induced metabolic alterations are commonly used for studying the function of SMs in plant stress responses (Szymański et al., 2020; Dussarrat et al., 2022). To reveal the stress adaptation strategy, the identification of metabolites that can distinguish between the tolerant and sensitive species is commonly applied (Hall et al., 2022). Based on the monitoring of metabolic markers with predictive value, and their unique combinations, the phenotyping provides a powerful tool for estimation of the extent of plant stress tolerance (Yadav et al., 2019).

In response to external stimuli, several fold increase of phenolic compounds was documented in *Hypericum* spp. culture systems like *in vitro* shoot cultures, cell suspensions or greenhouse-grown plants. Various biotic/abiotic stimuli (elicitors) were shown to redirect metabolism toward the enhanced accumulation of SMs including naphthodianthrones, acylphloroglucinols, xanthones, flavonoids, hydroxycinnamic acids, and other metabolites. Up to date, the elicitation potential of a broad spectrum of factors affecting the SMs accumulation was studied: i) herbivores (Sirvent et al., 2003); ii) microorganisms including endophytic fungi (Gadzovska-Simic et al., 2015b; Bálintová et al., 2019), pathogenic fungi (Sirvent and Gibson, 2002; Çirak et al., 2005; Gadzovska-Simic et al., 2012; Meirelles et al., 2013), or bacteria (Pavlík et al., 2007; Tusevski et al., 2017; Bálintová et al., 2019); iii) chemical elicitors, such as stress-signal molecules and growth regulators (Gadzovska et al., 2007; Pavlík et al., 2007; Coste et al., 2011; Gadzovska et al., 2013; Wang et al., 2015), simple sugars, or complex plant carbohydrates (Kirakosyan et al., 2000; Pavlík et al., 2007; Gadzovska-Simic et al., 2014; Gadzovska-Simic et al., 2015a; Bálintová et al., 2019), nanoparticles (Sharafi et al., 2013; Jafarirad et al., 2021); and iv) environmental abiotic stimuli including drought, irradiation, or temperature (Southwell and Bourke, 2001; Zobayed et al., 2006; Sooriamuthu et al., 2013). These results are summarized in **Supplementary Table 1** (**Supplementary Material S.M.1**).

### 2.1 Naphthodianthrones and acylphloroglucinols

Within the plant kingdom, the polyketide pathway-derived naphthodianthrones are synthesized essentially by some *Hypericum* spp. Among prenylated polyketides identified in the representatives of Guttiferae (Clusiaceae), the acylphloroglucinols with prenylation patterns are commonly isolated from the genus *Hypericum* (Crockett and Robson, 2011; Porzel et al., 2014).

The accumulation of naphthodianthrones is restricted to the glandular structures, so-called dark nodules, which are found in approximately 2/3 of *Hypericum* sections (Ciccarelli et al., 2001; Crockett and Robson, 2011). New mass spectrometry imaging (MSI)-based techniques confirmed co-localization of hypericin, pseudohypericin and their protoforms protohypericin and protopseudohypericin in the dark nodules, as the sites of their accumulation within the leaf. The naphthodianthrones are co-localized in these structures even with other anthraquinones and bisanthraquinones. Similarly, hyperforin, together with its analogues adhyperforin or hyperfirin, were co-localized in the translucent pale cavities (Kusari et al., 2015; Kucharíková et al., 2016; Rizzo et al., 2019; Revuru et al., 2020). Since only members of the sections belonging to clade core *Hypericum* produce hypericins (Nürk et al., 2013), the application of naphthodianthrones as potential stress-induced markers in metabolic phenotyping is limited. Nevertheless, numerous studies have been focused on improvement of the *Hypericum* spp. chemical composition by stimulating biosynthesis of hypericins and acylphloroglucinol derivatives using different biotic/abiotic elicitors.

In *Hypericum* plants, the increased accumulation of hypericins and hyperforin usually signalizes stressful environmental conditions associated with higher altitudes, including the intensity and quality of light, extreme temperatures, or temperature fluctuations (Zobayed et al., 2005; Odabas et al., 2009; Germ et al., 2010; Brechner et al., 2011; Rahnavard et al., 2012). For example, each 70 to 100 µmol m^-2^ s^-1^ PAR (photosynthetically active radiation) resulted in a 1.5-fold increase of hypericin content in *H. perforatum* shoot cultures (Briskin and Gawienowski, 2001). In greenhouse-grown *H. perforatum* plants, increasing light intensities from 803.4 to 1618.6 µmol m^-2^ s^-1^ stimulated continuous rise of hyperforin, hypericin and pseudohypericin contents (Odabas et al., 2009). In relation to light quality, the red light and UV-B stimulated the production of hypericin, pseudohypericin and hyperforin in *H. perforatum* and *H. retusum* (Nishimura et al., 2007; Brechner et al., 2011; Namli et al., 2014).

Under higher temperature stress, the ambiguous plant responses were reported. In most of the studies aimed at *H. perforatum*, the naphthodianthrone and hyperforin content was found to be positively influenced by increasing temperature up to 30 or 35°C (Couceiro et al., 2006; Zobayed et al., 2006; Odabas et al., 2009). On the other side, Yao et al. (2019) and Couceiro et al. (2006) did not observe any stimulatory effect of increasing temperature on either hypericin or hyperforin accumulation in this species. However, accumulation of these compounds differs in plant organs and age of culture. The content of hypericins and hyperforin was the highest at 35°C in the shoots, while in the flowers, the maximum was reached at 20°C or 25°C (Zobayed et al., 2005).

In response to low temperatures, the accumulation of hypericins predominantly depends on a cold stimulus. While the exposure of *H. perforatum* plants to low but above zero temperature was accompanied by unchanged or significantly lower amount of hypericins, the subfreezing temperature (-4°C) resulted in a 1.6-fold increase of hypericin content (Petijová et al., 2014; Bruňáková et al., 2015). In post-cryogenic regenerants of *H. perforatum*, *H. rumeliacum* and *H. tetrapterum*, more than a 3-fold increase of hypericin accumulation was documented and a remarkable 38-fold increase of hyperforin content was observed in *H. rumeliacum* (Petijová et al., 2014; Bruňáková and Čellárová, 2017). The stimulatory effect of cryogenic treatment can be appended to its complex abiotic stress nature comprising the dehydration of cells, crystallization of free water, mechanical wounds caused by ice particles and cold itself. However, accumulation of hypericins and phloroglucinols was negatively correlated with a single stressor represented by the acute drought or osmotic stress (Gray et al., 2003; Zobayed et al., 2003).

Relatively low stimulation of naphthodianthrones biosynthesis reaching 1.3 to 4-fold increase was seen in *Hypericum* spp. shoot cultures subjected to various chemical elicitors. In *H. perforatum* and *H. adenotrichum*, the elevated production of hypericins was achieved by the addition of polyethylene glycol (PEG) or sucrose to culture media (Pavlík et al., 2007; Yamaner and Erdag, 2013), and more than 3-fold increase of hypericins was reported in nanoperlite-treated *H. perforatum* shoot cultures (Jafarirad et al., 2021). In reaction to phytohormones, the accumulation of naphthodianthrones depended on the type, combination, and concentration of phytohormones including the adenine-type (BAP, 6-benzylaminopurine; Kin, kinetin; ZT, zeatin), or phenylurea-type (TDZ; thidiazuron) cytokinins (Liu et al., 2007; Karakas et al., 2009; Coste et al., 2011). The hypericins content also increased when the stress-signaling molecules salicylic acid (SA), jasmonic acid (JA) and methyl-jasmonate (MeJA) were used for elicitation of *Hypericum* shoot cultures (Sirvent and Gibson, 2002; Liu et al., 2007; Pavlík et al., 2007; Coste et al., 2011; Gadzovska et al., 2013).

For stimulation of plant secondary metabolism by the elicitors of biotic origin, pathogenic or symbiotic microorganisms are usually inactivated, and a mixture of lipopolysaccharides, peptidoglycans and other cell wall components are used. The stimulatory effects of biotic elicitors depend on the type, intensity, and duration of stimuli. The accumulation of naphthodianthrones increased in *Hypericum* shoots treated with pectin or dextran (Gadzovska-Simic et al., 2014), mannan, pectin and β-1,3-glucan (Kirakosyan et al., 2000; Yamaner and Erdag, 2013). The elicitors derived from endophytic fungal mycelia of *Thielavia subthermophila, Piriformospora indica, Fusarium oxysporum* and *Trichoderma crassum* induced a 1.5-fold increase of hypericins in several representatives of the sections *Hypericum* and *Oligostema* regardless the elicitor type, while a 2.3-fold higher accumulation of phloroglucinols was observed in *H. kouytchense* after the treatment with *P. indica* (Bálintová et al., 2019). The content of phenolic compounds including hypericins and hyperforin also increased upon inoculation of *Hypericum* plants with pathogenic fungi, such as *Colletotrichum gloeosporioides* (Sirvent and Gibson, 2002), *Diploceras hypericinum*, *Phytophthora capsici* (Çirak et al., 2005), or *Nomuraea rileyi* (Meirelles et al., 2013). In contrast to unchanged hyperforin content, higher amounts of hypericins were detected in *H. perforatum* seedlings infected with a mix of arbuscular mycorrhizal fungi (Zubek et al., 2012; Lazzara et al., 2017). The content of naphthodianthrones in *Hypericum* plants was also stimulated by inoculation with bacteria, for example the bacterial strains isolated from rhizosphere of *Nicotiana glauca* wild population (Mañero et al., 2012). The inactivated *Agrobacterium tumefaciens* culture promoted production of hyperforin in *H. perforatum* shoots, although hypericin accumulation declined (Pavlík et al., 2007). In the transgenic plants of *H. tomentosum* regenerated form hairy root cultures induced by *A. rhizogenes*-mediated transformation, higher content of hypericins in comparison with control plants was observed (Henzelyová and Čellárová, 2018). Similarly, higher amount of hypericins along with a 7-fold increase of hyperforin was observed in transgenic lines of *H. perforatum* plants (Tusevski et al., 2017). Additionally, hypericins and hyperforin content increased upon feeding with herbivores represented by the generalist feeders *Spilosoma virginica* and *S. congrua*, or *Spodoptera exigua* (Sirvent et al., 2003).

Being an integral part of acquired systemic plant responses in *Hypericum* spp., the increased accumulation of naphthodianthrones and acylphloroglucinols was shown to be linked with various biotic and abiotic stress stimuli. However, applying phototoxic pigments hypericin and pseudohypericin to examine a stress tolerance is limited as the accumulation can only be stimulated to a certain physiological level. When compared with untreated *Hypericum* plants, the relatively low increase of hypericins content, usually not exceeding a 5-fold, could be attributed to morpho-anatomical structure of the tissues, in which these compounds accumulate. Based on the morphometric leaf parameters of 12 *Hypericum* species, a cubic degree polynomial regression function was proposed for estimation of the biosynthetic capacity of *Hypericum* shoot cultures (Kimáková et al., 2018). Furthermore, Saffariha et al. (2021) designed several models to predict changes of hypericin content in relation to ecological and phenological factors.

### 2.2 Flavonoids and other phenolic compounds

Within plant polyphenols, flavonoids are the best-known metabolites with antioxidant properties. Through the inhibition of ROS generation, scavenging of free radicals, absorption of UV-wavelengths, and protection of cell membranes, these compounds have a fundamental role in plant photoprotection. Moreover, flavonoids were found to act as signal molecules involved in plant developmental processes, including the morphogenetic responses induced by various biotic/abiotic stimuli (Ferdinando et al., 2012; Brunetti et al., 2013; Petrussa et al., 2013; Chandran et al., 2019; DeLuna et al., 2020). Based on distinct substitutions in the basic skeleton containing three phenolic rings, namely two 6-carbon rings A and B linked by the central 3-carbon C ring, the flavonoids can be subdivided into several major subgroups including flavonols, flavones, flavanones, flavanonols, flavanols, anthocyanins, isoflavonoids and chalcones (Panche et al., 2016). Flavonoids are often linked to mono- or oligosaccharides (glucose, galactose, rhamnose, xylose, arabinose) and can be mono-, di-, or tri-glycosylated (Nabavi et al., 2020). Due to a great antioxidant capacity and photoprotection, the flavonols represented by dihydroxy B-ring-substituted quercetin-3-O-glycosides along with monohydroxy B-ring-substituted kaempferol-3-O-glycosides, are the most studied flavonoids (Shah and Smith, 2020).

In relation to environmental conditions, the phytochemical profiling revealed substantial differences in the accumulation of polyphenolic compounds, especially flavonoids and hydroxycinnamates (Tattini et al., 2004). In common, the enhanced accumulation of flavonols and anthocyanins usually indicates a stress exposure (Jan et al., 2021b). Based on the spectrum and abundance of stress-induced metabolites, the flavonoid profile can be used to distinguish between the stress-exposed plants and their unstressed counterparts. For example, the quercetin-3-O-glucuronide/glucoside and kaempferol-3-O-galactoside/glucoside represent specific metabolites displaying significantly higher relative content in water-deficient plants of *Vitis vinifera* when compared with the unstressed control group (Gago et al., 2017). Furthermore, the species-specific responses to abiotic stress including differences in the content of quercetin and its glycosides enable to distinguish even between related species like *Crataegus laevigata* and *Crataegus monogyna* (Kirakosyan et al., 2004). The variation of biosynthetically related metabolites is usually further modified by other abiotic stimuli (de Abreu and Mazzafera, 2005) or concomitant biotic stresses (Schenke et al., 2019). As an example, the variation of quercetin and rutin in water stressed *Hypericum brasiliense* plants depended on the temperature regime (de Abreu and Mazzafera, 2005).

In the genus *Hypericum*, flavonoids and hydroxycinnamic acids were shown to be the most prevalent phenolic compounds involved in antioxidant responses (Zdunić et al., 2017). The production of several flavonoid compounds and chlorogenic acid increased in *H. perforatum* adventitious roots cultured in media supplemented up to 70 g L^-1^ of sucrose resulting in osmotic stress (Cui et al., 2010); the levels of quercetin aglycone and its glycoside rutin increased in shoots and roots of water-stressed *H. brasiliense* (de Abreu and Mazzafera, 2005); and the acute drought stress applied on *H. perforatum* shoots resulted in higher accumulation of quercetin glycosides in floral tissues (Gray et al., 2003). The content of several flavonoids including quercetin, hyperoside (quercetin-3-D-galactoside), rutin (quercetin-3-rutinoside), and quercitrin (quercetin-3-L-rhamnoside), along with kaempferol, amentoflavone, apigenin-7-glucoside, and chlorogenic acid also raised with the increasing light intensity and temperature; based on the relationship between the culture conditions (e.g. temperature, light intensity) and phenolics accumulation, a mathematical model was proposed for the prediction of phenolics content in *H. perforatum* plants (Odabas et al., 2010).

**Figure 1:**
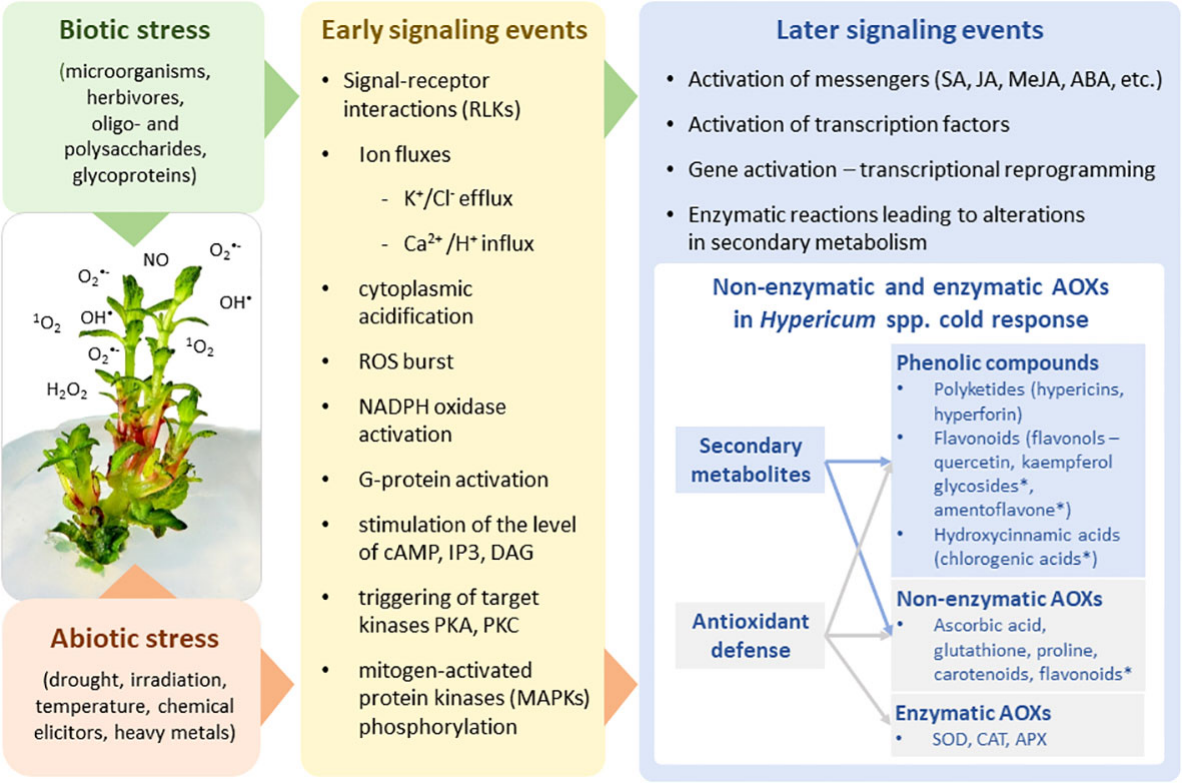
The biotic/abiotic stress signaling pathways comprising the stress perception, signal transduction, and expression of specific stress-related genes resulting in modulation of plant primary and secondary metabolism. Metabolites highlighted by an asterisk are the markers with predictive value in cold-stress response of *Hypericum* species.

A visible tissue coloring caused by the enhanced accumulation of flavonoids is usually taken as general indicator of physiological status of a stressed plant (Lu et al., 2017). Sooriamuthu et al. (2013) observed a 3 to 5-fold elevation of flavonoids and anthocyanins in photoinduced reddish-colored plantlets after transferring the etiolated *Hypericum hookerianum* shoots to the light. In *H. perforatum* shoot cultures, the reddening of plant organs due to accumulation of anthocyanins signalized the increased osmotic strength of culture medium after addition of sucrose (Pavlík et al., 2007). In our previous research (Bruňáková et al., 2011; Bruňáková and Čellárová, 2016), a purple pigmentation of leaf tissues was also seen in the post-cryogenic regenerants of *H. perforatum* (**Figure 1**).

The flavonoids and other phenolic compounds have been shown to strengthen the tolerance to a wide range of elicitors of biotic origin, including the pest and pathogen attacks. In our previous work, the changes in metabolic profiles of *Hypericum* spp. induced by glucose, starch, chitosan, and elicitors derived from *H. perforatum*-borne endophytic fungi were shown to be species-specific, depending on the type and intensity of stress stimuli (Bálintová et al., 2019). The content of rutin, hyperoside, isoquercetin (quercetin-3-glucoside) and quercitrin was elevated in all species regardless the elicitor, showing a maximum of 13-fold increase of isoquercetin in *H. monogynum* shoots treated with *P. indica-*derived elicitors in the presence of chitosan. On the other hand, the accumulation of amentoflavone and chlorogenic acid depended on the elicitation treatment and *Hypericum* species; the amentoflavone content raised maximally 15.7-times in *H. humifusum* shoots after culturing them on media supplemented with potato-dextrose broth (PDB), and a 13-fold increase was detected in *H. tetrapterum* after the treatment with elicitors derived from *T. subthermophila*, or *A. rhizogenes.* A maximum of 31.7-fold elevation of the chlorogenic acid was documented in *H. maculatum* shoots treated with D-glucose. Almost a 15-fold elevation of the chlorogenic acid was seen after the chitosan treatment in *H. kouytchense* shoots and a 11.5-fold increase of this metabolite was observed in *H. maculatum* treated by *A. rhizogenes-*derived elicitors (Bálintová et al., 2019).

The elevated accumulation of flavonols, hydroxycinnamic acids, xanthones, and other phenolic compounds stimulated by (poly)saccharides in *in vitro* cultures of *Hypericum* spp. proves the universal role of saccharides-based elicitation (Kirakosyan et al., 2000; Gadzovska-Simic et al., 2014; Gadzovska-Simic et al., 2015a). The elicitors derived from *Aspergillus niger* and *F. oxysporum* stimulated the biosynthesis of phenylpropanoids or flavonoid glycosides in several *Hypericum* spp. (Azeez and Ibrahim, 2013; Gadzovska-Simic et al., 2015b). Similarly, Franklin et al. (2009), and Tusevski et al. (2015) documented a significant elevation of xanthone biosynthesis in *H. perforatum* cell cultures stimulated by elicitors derived from *A. rhizogenes* and *A. tumefaciens*. An enhanced content of lignin and flavonoids was observed in *H. perforatum* cell wall biomass after *A. tumefaciens* elicitation (Singh et al., 2014). In common, the structural components of the cell wall of fungal or bacterial pathogens induce defense reactions in both, the intact plants or plant cell cultures *via* production of ROS, hypersensitive response, or production of SMs with antimicrobial effects (Angelova et al., 2006).

Melatonin represents another stress-responsive metabolite involved in antioxidant defense in several *Hypericum* representatives, e.g., *H. perforatum*, or *H. kouytchense* (Murch and Saxena, 2006; Nguyen et al., 2018; Chung and Deng, 2020; Khan et al., 2020). According to Zhou et al. (2021), the overexpression of *HpSNAT1* gene coding for serotonin N-acetyltransferase (SNAT), the key enzyme involved in melatonin biosynthesis resulted in more than 4-fold increase of melatonin content in response to high salt and drought treatment. In plants, melatonin was shown to contribute to the acquisition of tolerance against various abiotic/biotic stresses including cold (Han et al., 2017; Qari et al., 2022), or pathogen attacks (Lee et al., 2014).

To differentiate between stress tolerant and sensitive plants by metabolic phenotyping, the metabolite alterations induced by biotic/abiotic stimuli should be distinguished from a great naturally existing interspecific variability within this genus (Bálintová et al., 2019). Across the *Hypericum* spp., the spectrum and abundancy of SMs vary at the population, species and genotype levels (Çirak et al., 2008; Nogueira et al., 2008; Crockett and Robson, 2011; Çirak et al., 2014; Çirak et al., 2015; Çirak et al., 2016; Çirak and Radusiene, 2019; Carrubba et al., 2021), and usually changes during ontogenesis (de Abreu et al., 2004). Recent metabolomics studies reveal both the common and unique alterations in metabolic profiles of plants exposed to biotic/abiotic stresses related to polyphenolic compounds represented by chlorogenic acid, and flavonoids, namely the amentoflavone, quercetin or kaempferol glycosides.

## 3 Tolerance of *Hypericum* spp. to cold stress - the case study

The ability to withstand low temperatures represents a limiting factor of geographical distribution of the plant species. Plants adapt to under-zero temperatures through two main adaptation strategies based either on the tolerance, or avoidance responses. The ‘freezing tolerant’ (FT) species tolerate the extracellular ice formation that is accompanied by the dehydration stress; on the other side, the ‘freezing sensitive’ (FS) species prevent the formation of ice crystals in their tissues (Squeo et al., 1991; Sung et al., 2003). While the avoidance strategy predominantly relies on physical adaptations like lowering of the freezing point or supercooling, the tolerance mechanism is mainly associated with metabolic adaptations (Gusta and Wisniewski, 2013). In addition to this, the subjection of FT plants to low, but non-freezing temperatures, increases their ability to survive the otherwise lethal temperatures (below 0°C) through the process known as cold acclimation (Thomashow, 1999; Zandalinas et al., 2020). In FT species, the low but above-zero temperatures induce the higher resistance to freezing based on maintaining the functional photosynthetic apparatus and integrity of cell membranes (Ding et al., 2019; Pan et al., 2022).

To reveal the mechanism, by which the plants resist a potential freezing injury, the combined morphological, physiological, and biochemical data are widely applied. Within the genus *Hypericum*, several approaches were successfully applied to distinguish between FT and FS species, including the measurement of thermal properties, e.g. the freezing temperature of the leaves, the quantification of the extent of membrane disintegration, e.g. using the electrolyte leakage assay (LT50 values) (Petijová et al., 2014; Bruňáková et al., 2015), or the antioxidant profiling (Skyba et al., 2010; Danova et al., 2012). The changes of plant habitus, mesophyll parenchyma thickness, and chloroplast ultrastructure helped to find an association between the extent of freezing-induced tissue damage and predicted tolerance strategy in post-cryogenic regenerants of several *Hypericum* representatives (Georgieva et al., 2014; Stoyanova-Koleva et al., 2015).

One of the aims of the case study was to identify the profiling metabolites indicating the freezing tolerance strategy in 10 *Hypericum* species differing in their geographical distribution, and taxonomically classified to the sections *Ascyreia, Androsaemum, Bupleuroides, Hypericum, Oligostema, Myriandra, Webbia* and *Adenosepalum* (**Table 1**). The phenolic compounds, along with other non-enzymatic antioxidants, proline and carotenoids, as well as enzymatic activities of ROS-scavenging enzymes, superoxide dismutase (SOD), catalase (CAT) and ascorbate peroxidase (APX), were determined following the exposure of plants to 4°C (Bul'ková, 2018). Briefly, *Hypericum* plants cultured *in vitro* were used for comparative analyses of untreated (control) plants growing at 23 ± 2°C and plants, which were subjected to 4°C for 7 days. In the extracts of aerial plant parts, the content of phenolic compounds was analyzed by high-performance liquid chromatography (HPLC-DAD); the assessment of non-enzymatic and enzymatic antioxidant systems was done by spectrophotometric methods. Interspecific differences in phenolic composition as well as enzymatic and non-enzymatic AOXs related to cold response were evaluated by the principal component analysis (PCA) and hierarchical cluster analysis (HCA) (**Supplementary Material S.M.2**).

**Table 1:** Worldwide distribution of 10 *Hypericum* species used in the study.

Species	Section	Distribution	Ref.
*H. kouytchense*	III *Ascyreia*	1500 – 2000 m natural: China (Guizhou) cultivated: Europe, USA naturalized: New Zealand	Robson, 1985
*H. androsaemum*	V *Androsaemum*	lowland to 1800 m (in Iran) natural: Northwest Europe, Mediterranean to Iran introduced: Australia, New Zealand, Chile	Robson, 1985
*H. bupleuroides*	VIII *Bupleuroides*	640 – 2100 m natural: Northeast Turkey, Southwest Georgia	Robson, 2001
*H. perforatum*	IX *Hypericum*	10 – 2750 (China), 2780 (Tajikistan), 3150 m (Afghanistan) natural: Europe, Mediterranean, Asia Minor, Northwest India, Altai, China, Northwest Mongolia introduced: worldwide	Robson, 2002
*H. maculatum*	IX *Hypericum*	0 (West Scotland) – 2650 m (Southeast Switzerland) natural: Euro-Siberia from Northwest Europe to western Siberia, south from northern Spain to southwestern Siberia introduced: Canada (British Columbia)	Robson, 2002
*H. erectum*	IX *Hypericum*	450 – 2250 m (China), 100 – 2100 m (Japan) natural: Russia (Sakhalin), South Korea, Japan (Ryūkyū, Okinawa), Taiwan, southeastern China	Robson, 2006
*H. humifusum*	XIV *Oligostema*	0 – 1500 m (Morocco) natural: West and central Europe, Great Britain, southern Scandinavia, east to Belarus and Ukraine, south to Serbia and Croatia, central Italy, Sardinia, Spain, Portugal, Madeira, Azores, North Morocco introduced: South Africa, Australia, New Zealand, Chile	Robson, 2010
*H. kalmianum*	XX *Myriandra*	180 – 400 m natural: USA and Canada adjacent to the Great Lakes along the Ottawa River cultivated: Great Britain, Germany, Japan, Australia, USA	Robson, 1996
*H. canariense*	XXI *Webbia*	(20-) 180 – 900 (-1200) m natural: Canary Islands, Madeira naturalized: Hawaiian Islands, southern California	Robson, 1996
*H. annulatum*	XXVII *Adenosepalum*	1212 m (Sardinia), 300 – 1330 m (Balkans), 1050 – 1725 m (Arabia), 1600 – 3000 m (Ethiopia), 1100 – 2700 m (East Africa) natural: Sardinia, Balkan peninsula, Saudi Arabia, East Sudan, Ethiopia. East Uganda, Southwest Kenya, North Tanzania	Robson, 1996

### 3.1 Metabolic profiling of plants exposed to 4°C

**Figure 2:**
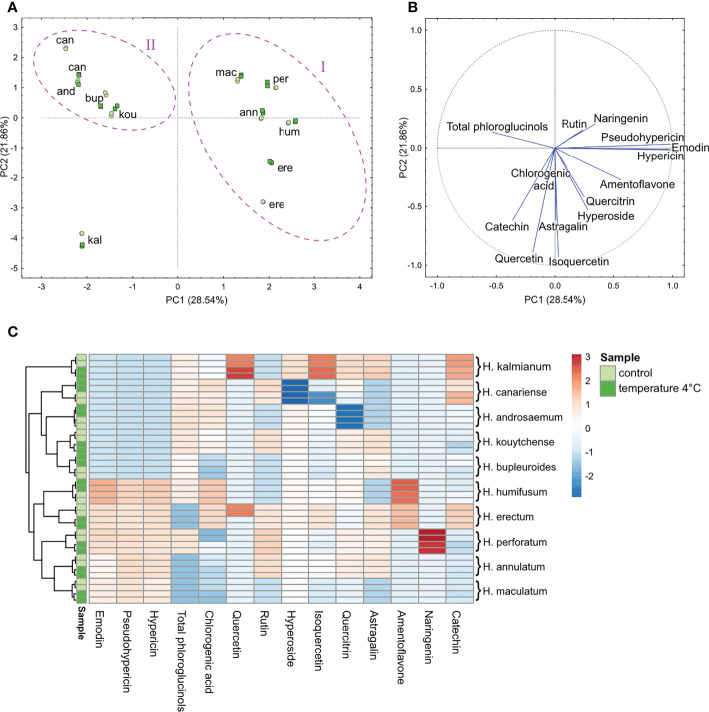
The PCA **(A, B)** and HCA **(C)** analyses of anthraquinones, phloroglucinols, chlorogenic acid and flavonoids quantified in the extracts of shoots of *in vitro-*grown *H.ypericum* spp. Clustering of species (cases) is highlighted with a magenta ellipse drawn around each cluster **(A)** in relation to metabolite contents (variables) **(B)**. Each species is represented by two biological replicates (two points per species). The HCA dendrogram **(C)** shows clustering of the species according to the branches. Each square in the heatmap dendrogram **(C)** represents the relative content of a metabolite. The red and blue color represent an increase and a decrease of relative metabolite content. Light green and dark green color of the samples indicate the metabolite contents in control plants and plants exposed to temperature of 4°C. (For interpretation of the references to color in this figure legend, the reader is referred to the Web version of this article.) kou, *H. kouytchense*; and, *H. androsaemum*; bup, *H. bupleuroides*; per, *H. perforatum*; mac, *H. maculatum*; ere, *H. erectum*; hum, *H. humifusum*; kal, *H. kalmianum*; can, *H. canariense*; ann, *H. annulatum*.

In control plants, the PCA revealed a great interspecific variability of *Hypericum* phenolic composition related to anthraquinones, acylphloroglucinol derivatives, flavonoids and chlorogenic acid (**Figures 2A, B**). According to the determinant metabolites, *Hypericum* representatives were distributed to two relatively compact groups and a singleton. The HCA-based heatmap (**Figure 2C**) was used to visualize the differences in the number of phenolic compounds between control plants and plants exposed to the cold treatment.

According to **Figure 2A**, more than 50% of the variance was accounted for the first two components PC1 and PC2. The species found in the 2nd and 4th quadrants formed a relatively compact group corresponding to cluster I, representing the sections *Hypericum* (*H. perforatum, H. maculatum, H. erectum*), *Adenosepalum* (*H. annulatum*) and *Oligostema* (*H. humifusum*). The remaining representatives seen in the 1st quadrant formed the separate cluster II comprising the sections *Webbia* (*H. canariense*), *Androsaemum* (*H. androsaemum*), *Bupleuroides* (*H. bupleuroides*) and *Ascyreia* (*H. kouytchense*). Based on the relative metabolite content, *H. kalmianum* (*Myriandra*) was the most distantly related species, forming a singleton in the 3rd quadrant. The HCA dendrogram confirmed PCA clustering showing two separate clades of hierarchically nested species and *H. kalmianum* as a separate branch (**Figure 2C**). Using both the PCA and HCA hierarchical heatmap, the determinant metabolites were identified for each cluster (**Figures 2B, C**). Naphthodianthrones and their potential precursor emodin were identified as the main determinants for grouping the species in cluster I. The phloroglucinol derivatives were the major profiling compounds of species belonging to cluster II. The flavonoid quercetin, and its glycoside isoquercetin, along with catechin and astragalin (kaempferol-3-O-glucoside), were the most abundant metabolites in *H. kalmianum*.

As we have shown previously, *H. kalmianum, H. perforatum* and *H. humifusum* were characterized as FT species; for example, *H. kalmianum* plants tolerated ice crystallization up to -11°C (Petijová et al., 2014). In the context of metabolic adaptations related to the freezing tolerance strategy, a relatively higher basal level of the flavonols quercetin and its glycosides, as well as astragalin in *H. kalmianum* suggests an interconnection between the flavonoids accumulation and plant ability to tolerate ice crystallization. Additionally, the elevated basal content of naringenin and amentoflavone was observed in other FT representatives, e.g., *H. perforatum* and *H. humifusum*. On the other side, no substantially elevated basal level of flavonoids was seen in *H. canariense* or *H. kouytchense*, which are known as FS species avoiding freezing by supercooling (Petijová et al., 2014).

In *Hypericum* plants exposed to 4°C, the HCA dendrograms did not reveal any substantial metabolic alterations excepting the enhanced quercetin content in *H. kalmianum* (**Figure 2C**). It should be noticed that the subjection of plants to 4°C for 7 days did not represent any lethal stress for *Hypericum* plants involved in this study. The plants did not change their habitus and continued their growth. However, the exposure of FT *Hypericum* spp. to temperature of 4°C for several days induced the processes typical for the cold acclimation (Bruňáková et al., 2011; Petijová et al., 2014). Under cold acclimation, a major reprogramming of the transcriptome, proteome, and metabolome results in the accumulation of various cryoprotective substances like saccharides, saccharide alcohols, low-molecular weight nitrogenous compounds, amino acids including proline, cold-regulated (COR) proteins, antioxidant enzymes, polyamines, and polyphenolic metabolites, including several subgroups of flavonoids (Fürtauer et al., 2019; Xu and Fu, 2022).

The relationship between the enhanced accumulation of flavonoids and plant tolerance to the under-zero temperatures is known and well documented. In the model species *Arabidopsis thaliana*, the flavonols, particularly kaempferols and quercetin glycosides, along with the anthocyanins, and phenylpropanoids were shown to be involved in the freezing tolerance by increasing the antioxidant capacity of tissues exposed to low temperatures (Schulz et al., 2015; Schulz et al., 2016; Zhang et al., 2019; Schulz et al., 2021). Apart from *Arabidopsis*, higher flavonoid contents, including flavonoid aglycones and glycosides, was associated with freezing tolerance in the number of species, for example *Primula malacoides* (Isshiki et al., 2014) or *Rhododendron* cultivars (Swiderski et al., 2004).

Despite the causal relationship between the accumulation of flavonoids and freezing tolerance has not been revealed yet, flavonoids have been successfully applied for prediction of the freezing strategy. For example, in *A. thaliana*, the unique combinations of these compounds were proposed as metabolic markers of the freezing tolerance (Korn et al., 2008; Schulz et al., 2016). The metabolic interactions between saccharides and plant SMs suggest a possible relationship between accumulation of flavonoid glycosides and enhanced tolerance to freezing in cold-acclimated plants (Korn et al., 2008; Fürtauer et al., 2019). Among the mechanisms, by which plant polyphenols contribute to the protection of plants against a freezing damage, the flavonoids and phenolic acids are directly involved in scavenging free radicals, modulation of the phytohormone-mediated stress responses, stabilization of the cell membranes, or preventing the proteins aggregation (Sharma et al., 2012; Hasanuzzaman et al., 2020).

On the other side, the molecular, biochemical, and physiological changes induced by cold acclimation lead to suppression of some subgroups of SMs accumulation in FT plants exposed to low but above-zero temperatures (Fürtauer et al., 2019). In our previous research, the subjection of FT *Hypericum* spp. to 4°C resulted in the decrease of some SMs, e.g., carotenoids and naphthodianthrones (Petijová et al., 2014; Bruňáková et al., 2015). Similarly, a decrease of terpene indole alkaloids content was seen in *Catharanthus roseus* plants under acclimated conditions (Dutta et al., 2013).

### 3.2 Antioxidant profiling of plants exposed to 4°C

**Figure 3:**
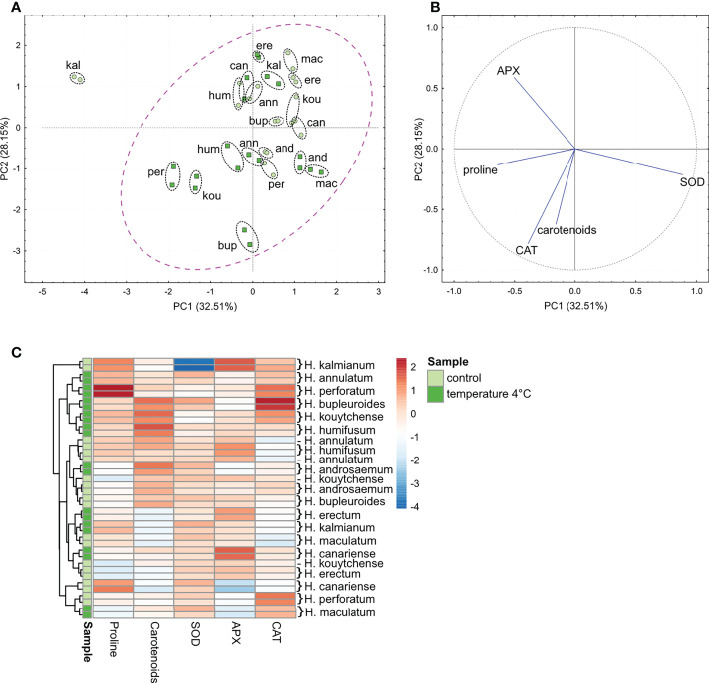
The PCA **(A, B)** and HCA **(C)** analyses of non-enzymatic (proline, carotenoids) and enzymatic (SOD, CAT, APX) antioxidant systems assessed in the extracts of shoots of *in vitro-*grown *H.ypericum* spp. A magenta-colored ellipse is drawn around the cluster of most of the species (cases) **(A)** in relation to content of non-enzymatic antioxidants, and activity of antioxidant enzymes (variables) **(B)**. Each species is represented by two biological replicates (highlighted with a black ellipse drawn around each pair of points per species). The HCA dendrogram **(C)** shows clustering of the species according to the branches. Each square in the heatmap dendrogram **(C)** represents relative content of a non-enzymatic antioxidants, or activity of antioxidant enzymes; an increase and a decrease is represented by the red and blue color. Light green and dark green color of the samples indicate the variables in control plants and plants exposed to temperature of 4°C. (For interpretation of the references to color in this figure legend, the reader is referred to the Web version of this article.) kou, *H. kouytchense*; and, *H. androsaemum*; bup, *H. bupleuroides*; per, *H. perforatum*; mac, *H. maculatum*; ere, *H. erectum*; hum, *H. humifusum*; kal, *H. kalmianum*; can, *H. canariense*; ann, *H. annulatum*.

The PCA and HCA revealed a naturally occurring interspecific variability in the basal level of non-enzymatic AOXs (proline and carotenoids), and enzymatic activities (CAT, SOD and APX). As shown by the PCA, more than 60% of the variability could be explained by PC1 and PC2 (**Figure 3A**). Within the control group of plants without the cold exposure, *H. kalmianum* formed a clearly separated singleton in the 1st quadrant based on the contribution of the profiling metabolites (**Figures 3A, B**). The other species were mostly situated in the 2nd quandrant (**Figure 3A**). According to the HCA dendrogram and HCA-based heatmap, the control plants of *H. kalmianum* represented a separate branch due to substantially higher basal level of APX activity. The cold-treated plants formed a common and relatively compact cluster spreading among all four quadrants (**Figure 3A**). The HCA heatmap revealed a substantial elevation of AOX enzymatic activities, along with the increased content of proline and carotenoids, which followed exposure of *Hypericum* plants to 4°C (**Figure 3C**).

#### 3.2.1 Non-enzymatic AOXs

In relation to the adaptation strategy, the cold exposure led to the highest proline accumulation in FT *H. perforatum*, while the carotenoids increased in the species exhibiting either the tolerance (*H. humifusum*) or avoidance defense mechanisms (*H. kouytchense, H. androsaemum*) (**Figure 3C**).

The relationship between non-enzymatic AOXs and freezing tolerance has not been fully understood yet. In common, proline is one of the main osmolytes contributing to the enhanced cold tolerance in plants (Verbruggen and Hermans, 2008). However, the content of proline does not indispensably correlate with the extent of freezing tolerance and depends on previous cold exposure of FT plants. The cytosolic proline increased in non-acclimated *A. thaliana* plants, but no correlation was found between the level of proline and elevated freezing tolerance in cold-acclimated plants (Hoermiller et al., 2022).

On the other hand, the level of carotenoids and ABA were shown to be in positive correlation with enhanced tolerance to cold stress e.g. in *Oryza sativa* ssp. *indica* possessing the tolerance strategy (Pan et al., 2022). Carotenoids are pigments, which act in essential physiological processes and plant antioxidant protection during various stresses. Being the important light harvesters, carotenoids have a crucial role in photoprotection of plants; they scavenge the ROS produced by triplet excited chlorophylls, dissipate the excess of absorbed energy during stress condition and stabilize the membranes (Uarrota et al., 2018). Besides, carotenoids are universal precursors of plant hormones including ABA that is known to be involved in hormone signal transduction in plants under cold conditions (Guo et al., 2018). However, a significant decrease of total carotenoids content was observed in FT *H. perforatum* or FS *H. canariense* plants exposed to the cold acclimation regime in our previous research (Bruňáková et al., 2015). In plants, the level of photosynthetic pigments depends on many factors; even in tolerant plants, the content of chlorophylls and carotenoids could be reduced in relation to the temperature decrease and duration of cold stress (Lukatkin et al., 2012). As an example, the temperature decline from 0°C to −4°C led to a partial reduction in the content of leaf carotenoids in *Vicia faba* tolerating freezing stress up to −10°C (Nabati et al., 2021). In sensitive plants, the cold stress usually leads to photosynthetic disruption (Wise and Naylor, 1987).

#### 3.2.2 Enzymatic AOXs

In *Hypericum* spp., we observed a substantial variability in the APX, CAT and SOD activities. Among the control plants, the highest basal activity of APX and CAT were seen in FT species *H. kalmianum* and *H. perforatum*, respectively (**Figure 3C**). The exposure of plants to a temperature of 4°C for 7 days led to various changes in antioxidant enzymatic activities in *Hypericum* representatives differing in the freezing adaptation strategies. The activity of SOD significantly elevated in *H. kalmianum* exhibiting strong freezing tolerance. The APX increased in *H. canariense* using avoidance strategy, and the CAT markedly rised in *H. bupleuroides* with the unknown mechanism of preventing the freezing injury.

Without the requirement of a cold acclimation, the naturally higher levels of enzymatic antioxidants might reflect the genetically predetermined stress response of plants under unstable environmental conditions. For example, the thylakoid membrane bound APXs (tAPXs) represent the extra-plastidic stress protection against a sudden decrease of temperature (van Buer et al., 2016). Coincidently, Chen et al. (2014) documented higher CAT activity in non-acclimated plants of *Chrysanthemum dichrum*, the species exhibiting a strong frost tolerance when compared with the weak frost resistant *Chrysanthemum makinoi.*

When the ambient temperature declines towards 0°C, plants use different enzymatic antioxidant systems to scavenge excessive ROS to minimize the oxidative stress in tissues (Xu et al., 2015; Karim and Johnson, 2021). Upon cold acclimation, the elevation of antioxidant enzymatic activities is well documented in the number of species that tolerate freezing, for example *Haberlea rhodopensis* (Georgieva et al., 2021), *Chrysanthemum* spp. (Chen et al., 2014), or *Eucalyptus* spp. (Oberschelp et al., 2020). Along with accumulation of osmolytes, the antioxidant enzymatic activities involving SOD, APX, and CAT, represent the main processes involved in plant tolerance to environmental stresses (Ahmad et al., 2010; Khaleghi et al., 2019).

The functional role of enzymatic AOXs in abiotic stress response of several *Hypericum* species has been well documented (Danova et al., 2012; Skyba et al., 2012; Georgieva et al., 2014). In *H. perforatum* tolerant to cryogenic treatment, a considerable intraspecific variability was seen related to the SOD, CAT, malondialdehyde (MDA), total free proline and carotenoids, suggesting the genotype-dependent differences in physiological and biochemical adaptations to the low-temperature treatment (Skyba et al., 2010).

In front-line of defense against ROS induced by cold stress, the SOD metalloenzymes represent the key enzymes controlling the oxidative status in higher plants (Sen Raychaudhuri and Deng, 2000). In plant cells, the SODs comprising the manganese (Mn-SOD), iron (Fe-SOD) and copper/zinc (Cu/Zn-SOD) catalyze the dismutation of O_2_^•-^ to less reactive products like O_2_ and H_2_O_2_ (Bueno et al., 1995; Alscher et al., 2002). The functional role of SOD in adaptation to cold stress conditions was reported for model plants like *Lycopersicum esculentum* (Soydam Aydin et al., 2013), *Nicotiana tabacum* (Wei et al., 2022), *A. thaliana* (Gill et al., 2010), or other plant species, for example a Himalayan high altitude alpine plant *Potentilla atrosanguinea* variety *argyrophylla* (Sahoo et al., 2001). According to Berwal and Ram (2018), the total SOD activity can be applied as an important biochemical marker indicating abiotic stress tolerance in higher plants. Moreover, the SOD isoenzyme profiles are commonly used as the selection criterion for screening plants for higher level of tolerance to various abiotic stimuli, including heat, cold, drought, salinity and heavy metal contaminants (Saed-Moucheshi et al., 2021).

The enzymatic defense including APX, and CAT contribute to an increased capacity of plant antioxidant tolerance to various environmental stresses (Yang et al., 2009; Yin et al., 2010). The APX and CAT are principal plant antioxidant H_2_O_2_-scavenging enzymes differing in their afinity to H_2_O_2_ and other organic peroxides (Sharma et al., 2012). In FT species *H. perforatum* and *H. kalmianum*, no increase in either CAT or APX activities was detected after exposure to the cold acclimation temperature of 4°C indicating that the CATs and APXs might represent an integral part of antioxidant defense in *Hypericum* species that acclimate after exposure to low but non-freezing temperatures (**Figure 3C**). It has been shown that the plant AOXs, such as the CATs normally operate below their maximum capacity, even if H_2_O_2_ concentrations reach relatively high values (Mhamdi et al., 2010). On the other side, when FS *Hypericum* plants were subjected to 4°C, higher CAT and APX activities were observed in *H. canariense* and *H. kouytchense*, respectively. An increase in CAT, APX or other antioxidant enzymatic activities might signalize the oxidative burst in plant tissues indicating the mechanism by which sensitive plants compensate higher level of ROS during cold exposure. For example, the H_2_O_2_ and MDA content increased more in the sensitive genotypes of *Hordeum vulgare* than in the tolerant ones (Valizadeh et al., 2017). However, the enhancement or depletion of H_2_O_2_-scavenging enzymes depends on several factors including the type, intensity, and duration of stress stimuli, which should be taken into consideration (Bayat et al., 2018).

### 3.3 Prediction of tolerance to cold stress – concluding remarks

In response to acclimation temperature of 4°C, the metabolic and antioxidant phenotyping revealed substantial alterations in basal level of phenolic compounds, along with other AOXs non-enzymatic components and enzymatic activities among *Hypericum* species differing in their geographical distribution. In relation to phenolic composition, the flavonols quercetin and quercetin glycosides, as well as astragalin, and other flavonoid metabolites like naringenin and amentoflavone could be considered as metabolites contributing to the freezing tolerance in the genus *Hypericum*. Higher basal level of proline, carotenoids and enzymatic activities of SOD, CAT and APX might reflect the genetically predetermined mechanisms of a stress response in tolerant species or signalize the oxidative burst in sensitive *Hypericum* representatives.

The tolerance to cold stress is a highly complex trait influenced by multiple factors; the reconfiguration of primary and secondary metabolic pathways along with activation of antioxidant enzymatic defense usually reflect the genetically predetermined capacity of the species adaptation to the local stress conditions. Based on electrolyte leakage (LT50 values), the freezing adaptation strategy was shown to be consistent with the natural habitat of a species. Within the genus *Hypericum*, the extent of the freezing tolerance was shown to concur with the post-cryogenic regeneration reflecting the geographical distribution of a species (Petijová et al., 2014; Bruňáková et al., 2015). While the (sub)tropical *H. canariense*, endemic of Canary Island and Madeira (Robson, 1996), was referred as cold-sensitive species avoiding ice formation by supercooling, other studied *Hypericum* species originating from the temperate zones, or growing at higher altitudes of the subtropical areas were shown to be relatively tolerant to freezing (Petijová et al., 2014). For example, the nearly cosmopolitan *H. perforatum*, and *H. kalmianum* restricted to cold area of USA and Canada (adjacent to Ontario Lake and Ottawa River) were characterized as the FT species (Petijová et al., 2014; Bruňáková et al., 2015). However, the prediction of freezing resistance strategy based on metabolic and antioxidant profiling is usually complicated by a large intraspecific variability related to the origin of the plant accession. The conflicting evidence of tolerance/avoidance mechanism was reported for *A. thaliana* accessions originated from different habitats (Zhen and Ungerer, 2008; Hoermiller et al., 2018). As shown by Nägele and Heyer (2013), the cold-induced intensive accumulation of saccharides and amino acids was higher in FT accessions of *A. thaliana* when compared with their FS counterparts. Significant alterations in AOX metabolism were also observed between FT and FS cultivars of *Hordeum vulgare* (Dai et al., 2009).

In conclusion, we assume that the combination of metabolic and enzymatic AOXs, along with other physiological markers including the thermal properties of plant tissues (LT50), and knowledge of the plant origin could be used for prediction of a mechanism, by which *Hypericum* spp. adapt to a cold stress. Albeit some classes of phenolic compounds contribute to the enhanced stress tolerance, more data are needed to identify the unique combinations of metabolites that would indirectly allow to estimate the extent of freezing tolerance of a particular species in the genus *Hypericum*.

## 4 Summary

Even though the genus *Hypericum* belongs to most diverse plant taxa, rare and endemic representatives might be threatened due to increasing climate changes contributing to the destruction of their natural habitats. In common, plant adaptation to changing environment depends on the activation of the cascades of molecular networks related to stress perception, signal transduction, induction of enzymatic activity and biosynthesis of SMs involved in the acquired systemic plant responses. Although the knowledge of AOX systems responsive to a particular stressor, such as low temperature, is critical, the assessment of a combination of several metabolic, physiological, and molecular markers is needed to understand the molecular mechanisms of the overall biotic/abiotic stress tolerance. The metabolic and antioxidant profiling of *Hypericum* spp. revealed a great species-dependent and environmentally influenced variability that usually complicates the application of phenotyping to estimate the plant stress tolerance. Therefore, the stress-induced changes in metabolic and antioxidant profiles should always be evaluated in a complex manner; in addition to the species-specific structural or physiological predeterminations, the applicability of profiling metabolites is also crucial. Within the genus, the most prominent stress-induced responses are usually associated with the accumulation of polyphenolic compounds including the chlorogenic acid, and flavonoids, namely the amentoflavone, quercetin or kaempferol glycosides, indicating their function in tolerance against various biotic/abiotic stresses. Although current mathematical models revealed contribution of genetic and environmental factors, more data are needed for relevant prediction of biosynthetic capacity of *Hypericum* spp. in relation to overall stress tolerance.

## Author contributions

KB summarized up-to-date knowledge on the topic, MB performed all the experiments and HPLC analysis of plant material. MB, KB, and LP statistically analysed and interpreted chemical data. MB, KB and LP prepared the figures and tables. EČ conceived the project and coordinated all stages of the experimental work. MB and KB drafted the manuscript and EČ revised and approved the manuscript. All authors contributed to the article and approved the submitted version.

## Funding

This work was supported by the Scientific Grant Agency (VEGA) of the Ministry of Education of Slovakia [grant number 1/0013/19] and the Slovak Research and Development Agency (APVV) [grant number APVV-18-0125].

## Acknowledgments

The authors wish to thankfully acknowledge RNDr. Viktória Bul'ková for her assistance with chemical analyses of the samples.

## Conflict of interest

The authors declare that the research was conducted in the absence of any commercial or financial relationships that could be construed as a potential conflict of interest.

## Publisher’s note

All claims expressed in this article are solely those of the authors and do not necessarily represent those of their affiliated organizations, or those of the publisher, the editors and the reviewers. Any product that may be evaluated in this article, or claim that may be made by its manufacturer, is not guaranteed or endorsed by the publisher.
