# Supplementary material for: Does phenotyping of Hypericum secondary metabolism reveal a tolerance to biotic/abiotic stressors?
Source: Front Plant Sci. 2022 Nov 30;13:1042375. doi: 10.3389/fpls.2022.1042375 (PMC9748567; doi:10.3389/fpls.2022.1042375)
Supplement: Supplementary file 1 [file DataSheet_1.docx]

**Supplementary material S.M.1**

**Table 1** Stress or elicitor-increased accumulation of defense-related secondary metabolites in *Hypericum* spp. shoot cultures.

| **Metabolites** | | **Stressors/Elicitors** | | | | | | | | | | ***References*** |
| --- | --- | --- | --- | --- | --- | --- | --- | --- | --- | --- | --- | --- |
|  |  | **Abiotic origin** | | | | | **Biotic origin** | | | | |  |
|  |  | ***High temperature***  (30 to 35°C) | **Low temperature**  (-4°C, cryogenic stress) | ***Light***  (e.g. intensity of light, red light, UVB) | ***Osmotic stress***  (e.g. water stress, addition of sucrose, or glucose, PEG, salt stress) | ***Chemical elicitors***  (nanoperlite, plant growth regulators, e.g. BAP, Kin, ZT, TDZ, SA, JA, MeJA) | ***Endophytic fungal mycelia*** (e.g. *T. subthermophila, P. indica, F. oxysporum* and *T. crassum*), treatment with D-glucose, or polysaccharides, e.g. chitosan, PDB) | ***Pathogenic fungi***  (e.g. *C. gloeosporioides*, *D. hypericinum,* *P. capsici, A. niger, N. rileyi)* | ***Mycorrhizal fungi***  (mix of arbuscular fungi Rhizophagus intraradices, Funneliformis constrictum, F. geosporum and F. mosseae) | ***Bacteria***  (e.g. *A. tumefaciens, A. rhizogenes*) | ***Feeding with herbivores***  *(e.g.S. virginica, S. congrua, S. exigua)* |  |
| Naphthodianthrones | Hypericin  Pseudohypericin | yes  (up to 3-fold) | yes (up to 3-fold) | yes | yes | yes  (up to 13.58-fold) | yes (up to 1.5-fold) | yes | yes | yes  (transgenic lines) | yes | Kirakosyan et al., 2000;  Briskin and Gawienowski, 2001;  Sirvent and Gibson, 2002;  Gray et al., 2003;  Sirvent et al., 2003  Zobayed et al., 2003;  Çirak et al., 2005;  Zobayed et al., 2005;  Couceiro et al., 2006;  Liu et al., 2007;  Nishimura et al., 2007;  Pavlík et al., 2007,  Karakas et al., 2009;  Odabas et al., 2009;  Germ et al., 2010;  Brechner et al., 2011;  Coste et al., 2011;  Mañero et al., 2012;  Rahnavard et al., 2012;  Zubek et al., 2012;  Gadzovska et al., 2013;  Meirelles et al., 2013;  Yamaner and Erdag, 2013;  Gadzovska-Simic et al., 2014;  Namli et al., 2014;  Petijová et al., 2014;  Bruňáková et al., 2015;  Bruňáková and Čellárová, 2017;  Lazzara et al., 2017;  Tusevski et al., 2017;  Henzelyová and Čellárová, 2018;  Bálintová et al. 2019;  Yao et al. 2019;  Jafarirad et al., 2021 |
| Acylphloroglucinol derivatives | Hyperforin | yes | yes (up to 38-fold) | yes | yes | yes (up to 6.16-fold) | yes  (up to 2.3-fold) | yes (slight increase) | na | yes (transgenic lines, up to 7-fold) | yes |  |
| Flavonoids | Quercetin aglycone and quercetin glycosides (rutin, hyperoside, isoquercetin, quercitrin) | yes | na | yes (up to 5-fold) | yes | na | yes  (up to 13-fold) | yes | na | yes  (in cell walls) | na | Gray et al., 2003;  de Abreu and Mazzafera, 2005;  Pavlík et al., 2007;  Germ et al., 2010;  Odabas et al., 2010;  Gadzovska et al., 2013;  Meirelles et al., 2013;  Sooriamuthu et al., 2013;  Namli et al., 2014;  Singh et al. 2014;  Tusevski et al., 2017;  Bálintová et al., 2019;  Yao et al. 2019 |
|  | Kaempferol aglycone and kaempferol glycosides (astragalin) |  |  |  | na | na | na | na | na | na | na |  |
|  | Amentoflavone |  |  |  | na | na | yes (up to 15.7-fold) | na | na | yes (up to 13-fold) | na |  |
|  | Apigenin-7-glucoside |  |  |  | na | na | na | na | na | na | na |  |
|  | Anthocyanins |  |  |  | yes | na | na | na | na | na | na |  |
| Hydroxycinnamic acids | Chlorogenic acids | yes | na | yes | yes | na | yes  (up to 31.7-fold) | yes | na | yes | na |  |
| Other metabolites | Melatonin | na | na | yes (up to 6-fold) | yes (more than  4-fold) | na | na | na | na | na | na | Chung and Deng, 2020;  Zhou et al., 2021 |

na – data not available

yes – stress-induced accumulation of secondary metabolites was recorde

**Supplementary material S.M.2**

**S.M.2: Material and Methods**

**S.M.2.1: Plant material and culture conditions**

*In vitro* grown shoots derived from stock cultures of 10 *Hypericum* species belonging to sections 3. *Ascyreia* (*H. kouytchense* H. Lév.); 5. *Androsaemum* (*H. androsaemum* L.); 8. *Bupleuroides* (*H. bupleuroides* Stef.); 9. *Hypericum* subsect. *Hypericum* ser. *Hypericum* (*H. perforatum* L., *H. maculatum* Crantz); 9. *Hypericum* subsect. *Erecta* (*H. erectum* Thunb.); 14. *Oligostema* (*H. humifusum* L.); 20. *Myriandra* subsect. *Centrosperma* (*H. kalmianum* L.); 21. *Webbia* (*H. canariense* L.); and 27. *Adenosepalum* (*H. annulatum* Moris) were used in the case study. The species identity was confirmed using molecular barcodes (^[[1]](#footnote-1)^Košuth *et al*. 2011; ^[[2]](#footnote-2)^Bruňáková *et al*. 2021).

The shoots were cultured on MS media (Duchefa Biochemie, Netherlands) containing salt mixture according to ^[[3]](#footnote-3)^Murashige and Skoog (1962) with Gamborg’s B5 vitamins (^[[4]](#footnote-4)^Gamborg *et al.* 1968), supplemented with 30 g·L^-1^ sucrose (CentralChem, Slovakia), 7 g·L^-1^ agar (REMI M. B., Czech Republic) and 2 mg·L^-1^ glycine. Before sterilization by autoclaving at 120°C and 120 kPa for 15 min, the pH of the media was adjusted to 5.6. Each plant growth vessel contained 30 mL of solid MS medium and 8 shoots that were subcultured every 5 weeks. The cultures were grown at 23 ± 2°C temperature under 16/8 h (day/night) photoperiod at 90 μmol·m^-2^·s^-1^ artificial irradiance.

**S.M.2.2: Cold treatment**

The four-week shoot cultures of each species were transferred to the thermostatic cabinet ET637-6 (Lovibond-Tintometer GmbH, Germany) for 7 days cultivation at 4°C. Simultaneously, four-week control plants were continuously kept at 23°C. The light and humidity conditions in both groups were the same. Two biological replicates that consisted of 3 culture vessels (24 shoots) per experimental/control group per species were used in the experiment.

**S.M.2.3: High-performance liquid chromatography**

The shoots were air dried at room temperature under shade conditions for a week and then in a dryer (BINDER, Germany) with 50 rpm outer air circulation at 40°C for 2 hours. Afterwards, the shoots were homogenised at 30 Hz by TissueLyser II homogeniser (Qiagen, Germany). 50 mg of homogenised plant material of each sample was extracted directly before chromatographic separations and the supernatant was collected into dark vials. The content of secondary metabolites was analysed by high-performance liquid chromatography (HPLC) using the Agilent 1260 HPLC system (Agilent Technologies, USA) equipped with DAD (diode array detector) and UV–Vis (ultraviolet–visible) lamp.

Anthraquinones and phloroglucinols were analysed according to ^[[5]](#footnote-5)^Tolonen *et al.* (2003) and modified by ^[[6]](#footnote-6)^Bruňáková and Čellárová (2016). Briefly, 1.5 mL of the extraction solution consisting of methanol (Sigma Aldrich, Germany) : ethanol (Merck, Germany) : acetone (Sigma Aldrich, United Kingdom) (1/1/1, v/v/v) was added to each sample. The samples were incubated for 30 min in ultrasonic bath (PS04000A Ultrasonic Compact Cleaner 4L Powersonic, Slovakia) at 25°C followed by centrifugation (U-32R Boeco) at 21,470 *g* and 20°C for 20 min. Extracts were separated by Agilent Poroshell 120 EC-C18 3.0×50mm 2.7 μm column (Agilent Technologies, USA) heated to 40°C with the injection volume of 10 μL. The mobile phase consisted of acetonitril gradient as follows: phase A, 10% acetonitril (ACN; CentralChem, Slovakia) with pH adjusted to 2.7 by trifluoroacetic acid (TFA; Sigma Aldrich, USA) mixed with phase B, 100% ACN, applied at flow rate of 1.3 mL·min^-1^. The starting ratio of phases was 80:20 (A:B) gradually changing to 20:80 in 8.5 min and to 0:100 in 9.5 min, thereafter returning to 80:20 in 16.7 min and held at this composition for 3.3 min giving the total analysis time of 20 min per sample.

The content of chlorogenic acid and flavonoids was measured as described by ^[[7]](#footnote-7)^Bálintová *et al*. (2019). Extraction was performed with 1.5 mL of 70% methanol and the extraction conditions were the same as for anthraquinones and phloroglucinols. The samples were separated by Kinetex C18 100 Å 150×4.6mm 5 μm column (Phenomenex, USA) heated to 30°C with the injection volume of 10 μL. The mobile phase was a mixture of phase A, 5% ACN (pH 2.7), and phase B, 80% ACN (pH 2.7), applied at flow rate of 0.9 mL·min^-1^. The gradient started at 100:0 (A:B) changing through 70:30 in 25 min to 0:100 in 30 min and held for 5 min followed by returning to 100:0 in 40 min.

The identification of individual metabolites was done by comparing the absorption spectra and retention times of each peak with the respective standards, and the quantity was determined according to the calibration curves as presented in ^2^Bruňáková *et al*. (2021). The anthraquinones were quantified based on hypericin calibration curve and represented as a sum of hypericin and protohypericin, and a sum of pseudohypericin and protopseudohypericin. The content of total pholoroglucinols was calculated as a sum of each peak representing hyperforin or related acylphloroglucinols with identical absorption spectra but different retention times.

**S.M.2.4: Non-enzymatic and enzymatic antioxidant systems**

The assessment of non-enzymatic and enzymatic antioxidant systems was done by spectrophotometric methods using spectrophotometer Uvi Light XTD2 (Secoman, France). For the analyses, only fresh shoots of experimental/control plants were used and the extraction took place directly after excision of plant material.

**S.M.2.4.1: Proline**

The content of proline was determined using modified protocol of ^[[8]](#footnote-8)^Carillo and Gibon (2011). Briefly, 1.5 mL of 50% ethanol (Centralchem, Slovakia) was added to 50 mg of fresh shoots and submerged in liquid nitrogen. Samples were homogenised and centrifuged at 21,470 *g* and 4°C for 20 min. 0.5 mL of the supernatant was mixed with 1 mL of the reaction solution containing 1% ninhydrin (Serva, Germany) dissolved in 60% acetic acid (Lach:ner, Czech Republic) and 20% ethanol. Blank consisted of 0.5 mL of 50% ethanol in 1 mL of the reaction solution. The mixtures and blank were incubated for 20 min in a water bath at 95°C followed by centrifugation at 10,950 *g* for 1 min. The absorbance was measured at 520 nm against blank. The quantification was made from calibration curve of L-proline (Sigma, USA) within the range from 0.2 mM to 5 mM proline.

**S.M.2.4.2: Carotenoids**

The content of carotenoids was measured according to ^[[9]](#footnote-9)^Lichtenthaler and Buschmann (2001). 1 g of fresh shoots was homogenised in the mortar with MgCO_3_ (Lachema, Czech Republic), sea sand (Penta chemicals, Czech Republic) and 80% acetone (Centralchem, Slovakia). Extract was filtered and fulfilled with 80% acetone to the total volume of 5 mL. The absorbance was measured at 435, 470, 515, 646 and 663 nm against blank (80% acetone). The content of carotenoids was calculated according to equation proposed by ^[[10]](#footnote-10)^Wellburn (1994) for 80% acetone as a solvent and 1 – 4 nm resolution range of spectrophotometer.

**S.M.2.4.3: The activity of enzymatic antioxidants**

Preparation of extracts for determination of the activity of enzymatic antioxidants followed the protocol of ^[[11]](#footnote-11)^Elavarthi and Martin (2010). 200 mg of fresh shoots were extracted with 1.2 mL of extraction solution (0.2 M phosphate buffer with 0.1 mM ethylenediaminetetraacetic acid (EDTA; Sigma, Germany); pH=7.8). The mixture was homogenised and centrifuged at 21,470 *g* and 4°C for 20 min. Supernatant was transferred to tube and pellet was extracted for the second time with 0.8 mL of extraction solution and centrifuged. The extracts were kept at -70°C until determination of enzymatic activity.

*S.M.2.4.3.1: Catalase (CAT)*

The activity of catalase was determined according to ^[[12]](#footnote-12)^Aebi (1984). The reaction mixture contained 2 mL of 200x diluted plant extract (chapter S.M.2.4.3) in 50 mM phosphate buffer (pH=7) and 1 mL of 10 mM H_2_O_2_ (Centralchem, Slovakia). The decomposition of H_2_O_2_ was followed as a decrease in absorbance at 240 nm for 3 min against blank consisting of reaction mixture without plant extract. CAT activity was calculated using molar extinction coefficient (ε) of hydrogen peroxide at 240 nm (40 mM^-1^·cm^-1^).

*S.M.2.4.3.2: Ascorbate peroxidase (APX)*

The ascorbate peroxidase activity was measured using protocol published by ^11^Elavarthi and Martin (2010). 1 mL of the reaction solution containing 50 mM phosphate buffer (pH=7) and 0.5 mM ascorbic acid (Sigma, China) was mixed with 10 µL of plant extract (chapter S.M.2.4.3). The decrease in absorbance was measured at 290 nm after addition of 50 µL of 10 mM H_2_O_2_ for 3 min against blank consisting of reaction solution. APX activity was calculated using molar extinction coefficient of ascorbic acid at 290 nm (2.8 mM^-1^·cm^-1^).

*S.M.2.4.3.3: Superoxide dismutase (SOD)*

Spectrophotometric determination of superoxide dismutase activity was done according to ^11^Elavarthi and Martin (2010). The reaction solution contained 50 mM phosphate buffer (pH=7), 2 mM EDTA, 9.9 mM L-methionine (Merck, Germany), 0.025% Triton X-100 (Amresco, USA) and 50 µM nitroblue tetrazolium (NBT; Serva, Germany). To start reaction, 40 µL of 2x diluted plant sample (chapter S.M.2.4.3) in 50 mM phosphate buffer (pH=7) was mixed with 2 mL of reaction solution and 20 µL of 1 mM riboflavin (Serva, Germany). The reaction solution mixed with riboflavin was used as blank. The samples and blanks were divided into two groups, one was illuminated by UV light for 30 min, and the other was kept in the dark. After 30 min, the absorbance was measured at 560 nm and activity of SOD was counted through percent inhibition of SOD and using molar extinction coefficient of formazan at 560 nm (14 mM^-1^·cm^-1^).

*S.M.2.4.3.4: Calculation of enzyme activity*

The activity of CAT and APX was calculated using the following equation:

**CAT/APX activity =**$\frac{\boldsymbol{\Delta A}}{\mathbf{t}}$**x**$\frac{\mathbf{1}}{\boldsymbol{\varepsilon}}$**x**$\frac{\mathbf{total reaction V}}{\mathbf{V of enzyme extract taken}}$**x**$\frac{\mathbf{total V of enzyme extract}}{\mathbf{g FW}}$ **x 1000**

ΔA: change in absorbance; ΔA= A_[3 min]_ - A_[0 min]_

t: incubation time of reaction; 3 min

ε: molar extinction coefficient denoted in mM^-1^·cm^-1^

V: volume

g FW: used plant material; g FW= 0.2 g FW of shoots

The activity of SOD was calculated using following equations:

**SOD % inhibition =** $\frac{\boldsymbol{sample light-sample dark}}{\boldsymbol{blank light-blank dark}}$ **x 100 = Y [%]**

sample/blank light: absorbance of samples/blanks after 30 min illumination by UV light

sample/blank dark: absorbance of samples/blanks after 30 min incubation in the dark

**1 unit of enzyme =** $\frac{\boldsymbol{1}}{\boldsymbol{50}}$ **x Y = Z [unit]**

**SOD activity =**$\frac{\mathbf{Z}}{\mathbf{30}}$**x**$\frac{\mathbf{1}}{\boldsymbol{\varepsilon}}$**x**$\frac{\mathbf{total reaction V}}{\mathbf{V of enzyme extract taken}}$**x**$\frac{\mathbf{total V of enzyme extract}}{\mathbf{g FW}}$ **x 1000**

**S.M.2.5: Statistical analysis**

The data obtained by HPLC and antioxidant system assessments were subjected to log-transformation and analysed by principal component analysis (PCA) using STATISTICA v. 7 package (StatSoft, USA). The dataset comprised variables represented by metabolite contents and antioxidant activities, control and cold acclimated *Hypericum* spp. were treated as cases. The results were visualized by two-dimensional score plots and correlation circles. The plots depicted the first two extracted principal components.

The studied species were grouped by hierarchical clustering analysis using web tool ClustVis ^[[13]](#footnote-13)^(Metsalu and Vilo, 2015). The dendrograms were constructed using Euclidean distance and complete linkage method. The differences between the samples were visualized by heatmaps. The color scales represented range of the standardized values.

1. Košuth, J., Smelcerovic, A., Borsch, T., Zuehlke, S., Karppinen, K., Spiteller, M., et al. (2011). The hyp-1 gene is not a limiting factor for hypericin biosynthesis in the genus *Hypericum*. Funct. Plant Biol. 38, 35–43. doi: 10.1071/FP10144 [↑](#footnote-ref-1)
2. Bruňáková, K., Bálintová, M., Henzelyová, J., Kolarčik, V., Kimáková, A., Petijová, L., et al. (2021). Phytochemical profiling of several *Hypericum* species identified using genetic markers. Phytochemistry. 187, 112742. doi: 10.1016/j.phytochem.2021.112742 [↑](#footnote-ref-2)
3. Murashige, T., and Skoog, F. (1992). A revised medium for rapid growth and bioassays with tobacco tissue culture. Physiol. Plantarum. 15, 473–497. doi: 10.1111/j.1399-3054.1962.tb08052.x [↑](#footnote-ref-3)
4. Gamborg, O. L., Miller, R. A., and Ojima, K. (1968). Nutrient requirements of suspension cultures of soybean root cells. Exp. Cell Res. 50, 151–158. doi: 10.1016/0014-4827(68)90403-5 [↑](#footnote-ref-4)
5. Tolonen, A., Hohtola, A., and Jalonen, J. (2003). Fast high-performance liquid chromatographic analysis of naphthodianthrones and phloroglucinols from *Hypericum perforatum* extracts. Phytochem. Anal. 14, 306–309. doi: 10.1002/pca.720 [↑](#footnote-ref-5)
6. Bruňáková, K., and Čellárová, E. (2016). Shoot tip meristem cryopreservation of *Hypericum* species. in Methods in Molecular Biology. Vol. 1391: Protocols for in vitro cultures and secondary metabolite analysis of aromatic and medicinal plants, ed. Mohan Jain S., (Springer Science + Business Media, New York, second edition), 31–46. doi: 10.1007/978-1-4939-3332-7_3 [↑](#footnote-ref-6)
7. Bálintová, M., Bruňáková, K., Petijová, L., and Čellárová, E. (2019). Targeted metabolomic profiling reveals interspecific variation in the genus *Hypericum* in response to biotic elicitors. Plant Physiol. Bioch. 135, 348–358. doi: 10.1016/j.plaphy.2018.12.024 [↑](#footnote-ref-7)
8. Carillo, P., and Gibon, Y. (2011). Protocol: extraction and determination of proline. PrometheusWiki01/2011. 1–4. [↑](#footnote-ref-8)
9. Lichtenthaler, H. K., and Buschmann, C. (2001). Chlorophylls and carotenoids: measurement and characterization by UV-VIS spectroscopy. Current Protocols in Food Analytical Chemistry*.* 1, F4.3.1–F4.3.8. doi: 10.1002/0471142913.faf0403s01 [↑](#footnote-ref-9)
10. Wellburn, A. R. (1994). The spectral determination of chlorophylls a and b, as well as total carotenoids, using various solvents with spectrophotometers of different resolution. J. Plant Physiol. 144, 307–313. doi: 10.1016/S0176-1617(11)81192-2 [↑](#footnote-ref-10)
11. Elavarthi, S., and Martin, B. (2010). Spectrophotometric assays for antioxidant enzymes in plants. in Plant Stress Tolerance, Methods in molecular biology. Vol. 639, ed. Sunkar R., (Humana Press), 273–280. doi: 10.1007/978-1-60761-702-0_16 [↑](#footnote-ref-11)
12. Aebi, H. (1984). Catalase *in vitro*. Method. Enzymol. 105, 121–126. doi: 10.1016/s0076-6879(84)05016-3 [↑](#footnote-ref-12)
13. Metsalu, T., and Vilo, J. (2015). ClustVis: a web tool for visualizing clustering of multivariate data using Principal Component Analysis and heatmap. Nucleic Acids Res. 43(W1), W566–570. doi: 10.1093/nar/gkv468 [↑](#footnote-ref-13)
